# Vitamin D deficiency and VDR gene polymorphism FokI (rs2228570) are associated with diabetes mellitus in adults: COVID-inconfidentes study

**DOI:** 10.1186/s13098-024-01328-6

**Published:** 2024-05-30

**Authors:** Samara Silva de Moura, Luiz Antônio Alves de Menezes-Júnior, Ana Maria Sampaio Rocha, Aline Priscila Batista, Thaís da Silva Sabião, Mariana Carvalho de Menezes, George Luiz Lins Machado-Coelho, Júlia Cristina Cardoso Carraro, Adriana Lúcia Meireles

**Affiliations:** 1 School of Nutrition, Postgraduate Program in Health and Nutrition, Research and Study Group on Nutrition and Public Health (GPENSC), Universidade Federal de Ouro Preto, Campus Morro do Cruzeiro, 35400- 000 Ouro Preto, Minas Gerais Brazil; 2 Epidemiology Laboratory, Medical School, Universidade Federal de Ouro Preto, Campus Morro do Cruzeiro, 35400-000 Ouro Preto, Minas Gerais Brazil; 3 Postgraduate Program in Biological Sciences, Universidade Federal de Ouro Preto, Campus Morro do Cruzeiro, 35400-000 Ouro Preto, Minas Gerais Brazil; 4 Department of Clinical and Social Nutrition, Research and Study Group on Nutrition and Public Health (GPENSC), School of Nutrition, Universidade Federal de Ouro Preto, Campus Morro do Cruzeiro, 35400- 000 Ouro Preto, Minas Gerais Brazil; 5 R. Diogo de Vasconcelos, 122, Ouro Preto, MG Brazil

**Keywords:** Single-nucleotide polymorphism, Vitamin D receptor, Population Genetics, Diabetes, Glycated hemoglobin

## Abstract

**Background:**

Diabetes mellitus is a chronic and multifactorial condition, including environmental risk factors such as lifestyle habits and genetic conditions.

**Objective:**

We aimed to evaluate the association of *VDR* gene polymorphism (rs2228570) FokI and vitamin D levels with diabetes in adults.

**Methods:**

Cross-sectional population-based study in adults, conducted from October to December 2020 in two Brazilian cities. The outcome variable was diabetes, defined as glycated hemoglobin ≥ 6.5% or self-report medical diagnosis or use of oral hypoglycemic drugs. Vitamin D (25-hydroxyvitamin D) was measured by indirect electrochemiluminescence, and classified as deficiency when 25(OH)D < 20 ng/mL. All participants were genotyped for VDR FokI polymorphism by qPCR and classified as homozygous mutant (ff or GG), heterozygous (Ff or AG), or homozygous wild (FF or AA). A combined analysis between the FokI polymorphism and vitamin D levels with diabetes was also examined. A directed acyclic graph (DAG) was used to select minimal and sufficient adjustment for confounding variables by the backdoor criterion.

**Results:**

The prevalence of DM was 9.4% and vitamin D deficiency (VDD) was 19.9%. The genotype distribution of FokI polymorphism was 9.9% FF, 44.8% Ff, and 45.3% ff. It was possible to verify a positive association between vitamin D deficiency and DM (OR = 2.19; 95% CI: 1.06–4.50). Individuals with the altered allele (ff) had a 1.78 higher prevalence of DM (OR: 1.78; 95% CI; 1.10–2.87). Combined analyses, individuals with vitamin D deficiency and one or two copies of the altered FokI allele had a higher prevalence of DM (Ff + ff: OR: 1.67; 95% CI; 1.07–2.61; ff: OR: 3.60; 95% CI; 1.40–9.25).

**Conclusion:**

Our data suggest that vitamin D deficiency and *FokI* polymorphism are associated with DM.

**Graphical abstract:**

**Supplementary Information:**

The online version contains supplementary material available at 10.1186/s13098-024-01328-6.

## Highlights

The prevalence of vitamin D deficiency was higher in patients with diabetes mellitus;

The FokI polymorphism was associated with DM and, in synergism with vitamin D deficiency, increased the likelihood of DM in adults.

The FokI polymorphism was the most frequent in individuals with diabetes;

Individuals with VDD were more predisposed to have DM regardless of phenotype, meaning that vitamin D deficiency is a relevant predictor for developing DM, regardless of whether or not there is an alteration in the VDR receptor.

**Supplementary Information:**

The online version contains supplementary material available at 10.1186/s13098-024-01328-6.

## Graphical abstract

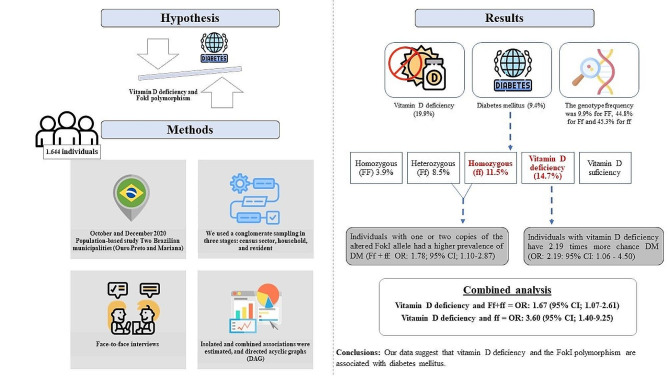

## Introduction

Diabetes mellitus is a chronic, multifactorial pathogenesis, in which genetic and environmental factors, as well as lifestyle habits, contribute to its development and complications [1]. he incidence of DM has been alarming, with a steep increase in prevalence reaching pandemic levels [2]. According to the World Health Organization, about 425 million people worldwide are affected by this condition [3], becoming a global health concern, given its deleterious effects on individual’s health and its high cost of management.

Given the characteristic of complications and the increasing prevalence of DM, there is an effort to understand the main causes and innovative approaches to contribute to its prevention and management. Specifically, in recent studies, an association between DM (type 1 and 2) and vitamin D deficiency (VDD) has been proposed [4–6]. I In the last two decades, a range of studies has been produced on its physiology, since its receptors have been found in different tissues and organs of the human body [7, 8]. Vitamin D deficiency has been observed to be a predictor for susceptibility to several pathophysiologies, such as cognition and mental health (anxiety, depression, and stress), insomnia, cardiovascular diseases, osteoporosis, infections, and some cancers [9–12]. In addition, vitamin D plays immunomodulatory, anti-inflammatory, antioxidant, and antifibrotic properties [13].

Recent studies show a potential therapeutic role in the immunomodulatory properties of vitamin D and its importance in glycemic metabolism [6]. One possible mechanism that explains this association is that vitamin D is a micronutrient involved in several reactions responsible for increasing insulin synthesis and clearance from β cells. In addition, vitamin D has been shown to have a compensatory effect in correcting hyperglycemia by causing increased insulin receptor expression, optimizing its sensitivity, and suppressing pro-inflammatory cytokines, which may favor insulin resistance [14]. Indirectly, it acts in maintaining normal concentrations of calcium flux in pancreatic β-cells (changes in this flux may present effects on insulin secretion and cytokine-induced apoptosis in pancreatic β-cells) [15–17]. Although it is obtained through food intake, most of the production of this micronutrient is by skin synthesis, due to sun exposure (ultraviolet B rays). Therefore, populations with reduced sun exposure have an increased risk of developing VDD, which is a global public health problem affecting children, adolescents, postmenopausal women, adults, and the elderly [17].

Intricating this scenery, the genetic part of the individual may play an important role in this association between VDD and DM. The action of vitamin D is mediated by its binding to its receptor (VDR) [5]. Several polymorphisms have been identified in the VDR gene, including FokI. These polymorphisms are believed to be the reason for the hereditary dysfunction of VDR [18]. Thus, studies have sought to elucidate the mechanisms involved in the association of FokI polymorphism and DM, however, investigations are still inconclusive, emphasizing the need for understanding in different ethnic populations.

Given that pancreatic beta cells have a wide distribution of vitamin D receptors [4], it is possible that the presence of polymorphisms in this receptor exerts influence on the relationship between vitamin D deficiency and diabetes. Therefore, the investigation of this study aims to verify whether vitamin D deficiency and the FokI polymorphism are associated with DM, alone and in combination. The hypothesis is that both are associated, and when combined the odds ratio increases.

## Methods

### Study design and sample size

This is a population-based seroepidemiological household survey conducted between the months of October and December 2020 in two Brazilian municipalities (Ouro Preto and Mariana), in the south-central region of Minas Gerais state.

We used a conglomerate sampling in three stages: census sector, household, and resident. The census sectors were considered as primary sampling units, selected with probability proportional to the number of households, using as a measure of size the number of households, obtained from the synopsis of the 2010 census of population [19]. Before choosing the primary units, prior stratification was performed, considering the average income, according to data from the 2010 demographic census of the Brazilian Institute of Geography and Statistics, to avoid the risk of drawing a sample from non-representative sectors. Therefore, the representativeness of the different socioeconomic strata (< 1 minimum wage, 1 to 3 minimum wages, and ≥ 4 minimum wages) was guaranteed in the final sample.

The secondary sampling units were the households, selected systematically using the updated listing of existing household units in the primary sampling units (selected census sectors). The household units are formed by private households with residents. After the selection of the census sectors, the household selection interval (k) was calculated for the systematic sampling, according to the formula: k = Ni / (xi/ni), where: Ni = total number of households in the census sector; xi = sample size; ni = number of households to be selected in the census sector. In this way, a proportional number of homes per sector was obtained, covering the entire geographical area. The first household of the census sector was selected according to Brazilian Institute of Geography and Statistics indications and, subsequently, the systematic sampling of the next household was done according to the household selection interval (k).

The tertiary sampling units were the individuals, selected from a simple random sampling. In the selected household, a list of all adult residents was made, and a simple random drawing of one resident to participate in the research was carried out.

The sample size was calculated using the OpenEpi program (https://www.openepi.com/Menu/OE_Menu.htm), totaling a minimum of 732 interviews for each municipality. During the data collection process, we evaluated 1,762 individuals, of which 25-hydroxyvitamin D and glycated hemoglobin were not analyzed in 71 due to insufficient blood samples, and 47 were not analyzed due to insufficient samples for extraction of viable DNA for genotyping. Therefore, for this study, 1,644 individuals were included, representing adult residents in the urban areas of the two cities.

This design was based on large national household surveys, such as the National Household Sample Survey (PNAD) [20], Family Budget Survey (POF) [21]; “Saúde em Beagá” survey [22], and more recently the “EPICOVID19” study [23].

The inclusion criteria for the study were adults (aged 18 years and older) with permanent residence in the urban areas of the municipalities, cognitive ability, and venous access to serological testing. The exclusion criteria were residents of community services and long-stay institutions who did not meet the inclusion criteria.

Face-to-face interviews were conducted in the participants’ homes using an electronic form by the interviewer. The questionnaire was subdivided according to sociodemographic and economic aspects, living habits, general health conditions, and quality of sleep. This study followed reported guidelines dictated by the Strengthening the Reporting of Observational Studies in Epidemiology (STROBE).

### Biological sample

Blood collection was performed by a trained professional, by puncture in the region of the cubital fossa. Two tubes were used: a 7.5 mL S-Monovette® (Sarstedt) serum gel tube for vitamin D analysis; and a 2.7 mL S-Monovette® (Sarstedt) containing sodium fluoride/EDTA for molecular biology. Subsequently, the samples were taken to the Laboratory of Epidemiology (LEPI) of the Medical School of the Federal University of Ouro Preto (UFOP). In the laboratory, the serum tubes were centrifuged at 2500 rpm for 15 min, aliquots were prepared, and stored in a -80 °C freezer until the vitamin D analysis. Samples containing EDTA were stored at -20 °C until the genetic analyses were performed.

### Outcome: diabetes mellitus

Glycated hemoglobin (HbA1c) was measured using the immunoturbidimetry method in the COBAS INTEGRA 400 plus automatic analyzer (Roche, Germany), following a protocol standardized by the manufacturer. Before each analysis, the device was calibrated with quality controls (HbA1c Control N and HbA1c Control P, Roche). A minimum volume of 400 µL of whole blood was used for the samples. To describe HbA1c levels, the cut-off points of < 5.70, 5.70–6.49, and > 6.50% were used [24].

Furthermore, we evaluated self-reported medical diagnoses of diabetes and medication use. Medications were classified according to the ATC-Anatomical Therapeutic Chemical, a system of alphanumeric codes developed by the World Health Organization (WHO) for the classification of drugs and other medical products [25]. Therefore, subjects were classified with diabetes if they had HbA1c levels ≥ 6.5% or a medical diagnosis of diabetes or used any medication in ATC class code A10 (medications used in diabetes) [24].

### Exposures variables: vitamin D and VDR gene FokI polymorphism

Vitamin D was determined by indirect electrochemiluminescence with competition principle in the Access 2 Immunoassay System® (Beckman Coulter, USA) with a Roche Diagnostics® commercial kit (Roche, Switzerland). For intra-laboratory analysis, the coefficient of variation of the method ranges from 6.1 to 7.5%, and the correlation coefficient with LC-MS/M was 0.92 (data provided by the manufacturer). Furthermore, in previous studies, this method was performed against LC-MS/MS [26], and LC-MS/MS, in turn, was standardized against the NIST standard [27]. Vitamin D concentrations were classified as a deficiency according to the Institute of Medicine (IOM) as “deficient” when 25(OH)D < 20 ng/mL; and “sufficient” when 25(OH)D ≥ 20 ng/mL [28].

The genomic DNA extraction was performed with Wizard® Genomic DNA Purification kit (Promega, USA) according to the manufacturer’s protocol. After extraction, the DNA was maintained for 24 h in a hydration solution at a temperature of 4 °C and then dosed by fluorimetry (Qubit 2.0 Fluorometer, Invitrogen®). The DNA samples were stored at -20 °C until the moment of their analysis.

The allelic discrimination of the FokI polymorphism (rs2228570 A > G) in the VDR gene, which consists of a nucleotide base change from adenosine (A) to guanine (G). The analysis was performed by the real-time PCR (qPCR) technique using the TaqMan® SNP Genotyping Assay System (Applied Biosystems, Foster City, USA), consisting of fluorescently labeled (FAM and VIC) probes (Applied Biosystems, Foster City, CA) in the 7500 Fast Real-Time PCR Systems equipment (Applied Biosystems, USA), according to the manufacturer’s instructions [29]. Participants were classified as homozygous mutant (ff, also called GG), heterozygous (Ff, also called AG), or homozygous wild (FF, also called AA).

### Covariates

The sociodemographic and economic variables evaluated were sex (female or male), age group (18–34; 35–59; ≥ 60 years), marital status (single or married), current family income (≤ 2; > 2 to ≤ 4; > 4 minimum wages), an education level (< 8; 9–11; ≥ 12 years of study). Self-reported skin color was evaluated using the categories proposed by the Brazilian Institute of Geography and Statistics (IBGE) [30], and they were categorized into white, black/brown, and other skin colors (indigenous and yellow).

Health conditions evaluated were current smoking (yes or no), current alcohol drinking (yes or no), physical activity (activity when they reached at least 150–300 min of moderate-intensity aerobic physical activity, or at least 75–150 min of vigorous-intensity aerobic physical activity per week, or inactivity when the recommendations were not reached) [31]. Nutritional status was evaluated by body mass index (BMI), from self-reported height (cm) and weight (kg). BMI was classified as underweight (BMI < 18.5 kg/m2 if aged < 60 years; BMI < 23.0 kg/m2 if aged ≥ 60 years), eutrophic (BMI 18.5–24.9 kg/m2 if aged < 60 years; BMI 23.0–28.0 kg/m2 if aged ≥ 60 years), overweight (BMI 25.0–29.9 kg/m2 if aged < 60 years; BMI 28.0–29.9 kg/m2 if aged ≥ 60 years), and obesity (BMI > 30.0 kg/m2) [32, 33]. In addition, we assessed the diseases related to vitamin D metabolism, which were self-reported by the individuals (chronic obstructive pulmonary disease, chronic kidney disease, cancer, cardiovascular disease or thyroid disease), assessed separately and dichotomized into (presence or absence).

Exposure to daily sunlight was assessed quantitatively, from the following questions: “From Monday to Sunday, how many times a week, and for how long are you exposed to the sun?“. Subsequently, the average daily sunlight was calculated from the following formula: [weekly frequency of sunlight (0 to 7 days) x daily time of sunlight (minutes) / 7] and classified as dichotomous; insufficient sun exposure (< 30 min per day) and sufficient sun exposure (> 30 min per day) [34].

Moreover, we evaluated whether individuals used any vitamin D dietary supplements by self-reporting, “In the past three months, have you used a vitamin-based dietary supplement, such as vitamin D or cholecalciferol or cod oil supplementation?” (yes or no).

### Statistical analysis

Initially, the sample weight was calculated to adjust the natural weights of the sampling design and/or correct problems caused by the absence or refusal to answer, assigning different weights to the sample elements, corresponding to the inverse of the product of probabilities used in the selection stages [35].

Categorical variables were described as relative frequencies and 95% confidence interval (95% CI), and continuous variables were described as means and 95% CI. All statistical analyses were performed considering the study design and sampling weighting factors using the “svy” package of Stata® software, version 15.0. The significance level was set at 0.05.

Allele frequencies were estimated with the gene counting method. Departure from Hardy–Weinberg equilibrium (HWE) was estimated by an exact two-sided probability test using the formula provided by Weir [36].

**Fig. 1:**
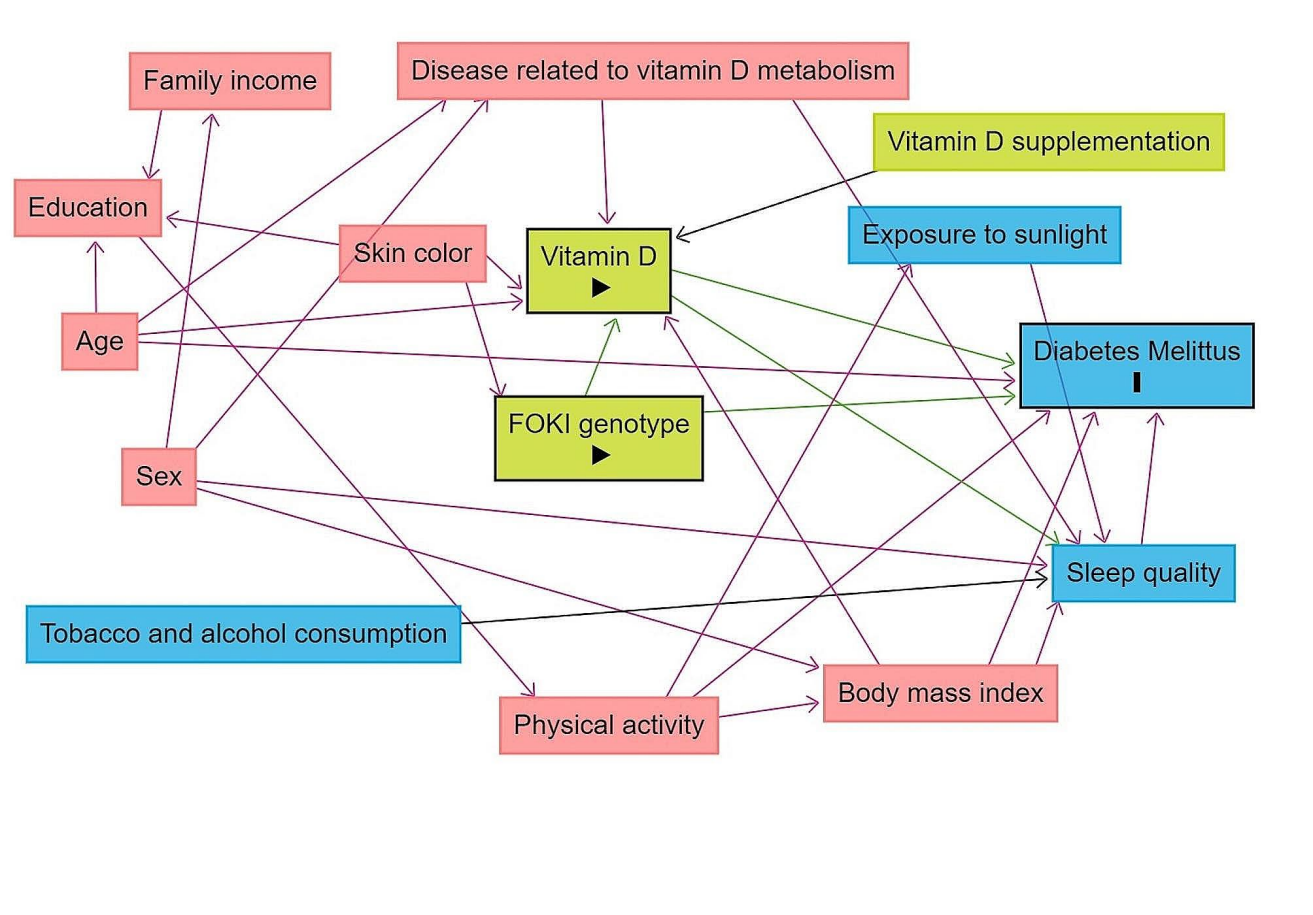
Directed acyclic graph (DAG) of the association between FokI polymorphism and vitamin D with diabetes in adults Subtitle: The variable in green and with the “►” symbol inside the rectangle was the exposure variable; those in blue and with the letter “I” inside the rectangle were the response variables; variables in blue are the antecedents of the outcome variable; and those in red are antecedents of the outcome and exposure variables

Furthermore, a theoretical model based on a directed acyclic graph (DAG) was developed according to the exposure variable (vitamin D and FokI polymorphism), outcome (diabetes mellitus), and covariates, using the online software Dagitty, version 3.2. Causal connections represented by arrows were established between variables (Fig. 1). To avoid unnecessary adjustments, spurious associations, and estimation errors, the backdoor criterion was used to select a minimum set of confounding variables to fit the analyses [37]. Hence, the model was adjusted by the following minimum and sufficient set of variables: age (continuous variable), sex (male or female), family income (≤ 2; > 2 a ≤ 4; > 4 minimum wages), body mass index (continuous variable, kg/m²), disease related to vitamin D metabolism and physical activity (physically active or physically inactive).

In addition, separate associations between the FokI polymorphism and VDD in the outcome of DM were proposed, and then multiplicative combined analysis was performed between the FokI + VDD polymorphism in the association with the outcome (DM). The adjustments cited above were maintained for these analyses.

Unadjusted and adjusted logistic regressions were performed for the variables indicated by DAG. The variance inflation factor assessed collinearity between covariates with the “subsetByVIF” package considering a maximum cutoff point of 10 (VIF < 10) [38, 39].

### Ethical considerations

All procedures followed Brazilian guidelines and standards for research involving human beings of the Declaration of Helsinki and were approved by the Research Ethics Committee (Ethics Submission Certificate No. 32815620.0.1001.5149).

## Results

### Population description

Table 1 shows the sociodemographic characteristics and health conditions of the study participants. Of the participants, 51.9% were women, the most prevalent age group was 35 to 59 years (45.6%), most were married (54.0%), had from 9 to 11 years of education (39.7%), had a family income equal or less than two minimum wages (43.0%).

Concerning health conditions, 52.3% of the individuals had poor sleep quality, consumed alcohol (58.3%), were overweight (37.0%) or obese (18.6%), and were physically inactive (69.7%).

### Vitamin D

The mean of vitamin D was 26.2 ng/mL (95%CI: 25.2–27.1), and the prevalence of vitamin D deficiency was 19.9% (95%CI: 15.6–25.0) (Table 1).

### Diabetes Mellitus

**Fig. 2:**
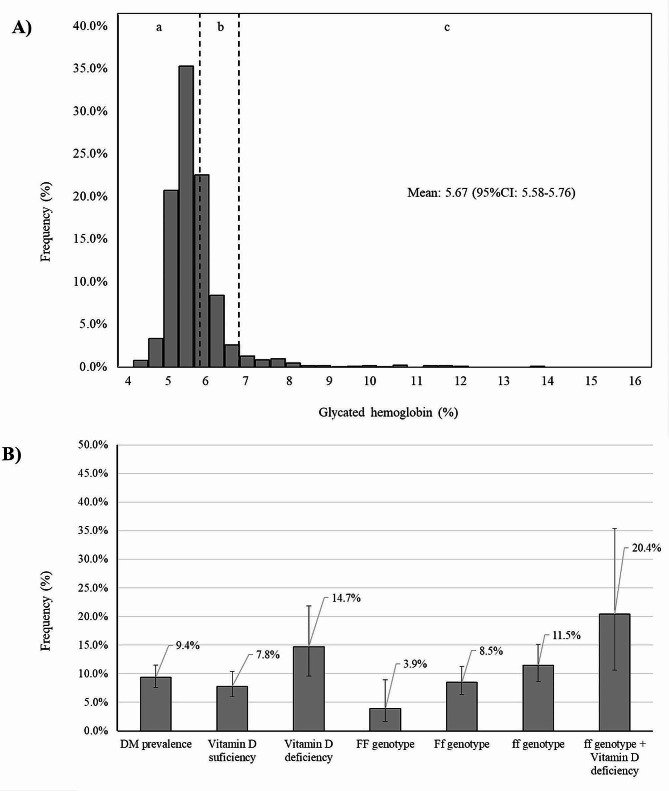
Distribution of glycated hemoglobin levels (%) (Fig. 2a) and the frequency of diabetes mellitus according to FokI genotype and Vitamin D status (rs2228570) in adults (Fig. 2b) Subtitle: a– values < 5.70 (64.7%); b– values 5.70–6.49 (28.6%); c– values ≥ 6.50 (6.70%)

Mean HbA1c values were 5.6% (95% CI: 5.58–5.76). Thus, 64.7% of subjects had values HbA1c values < 5.7, 28.6% had HbA1c values between 5.7 and 6.5, and 6.7% values > 6.5% (Fig. 2a). Furthermore, 6.8% reported a medical diagnosis of diabetes (95%CI: 5.3–8.7) and 1.9% used some diabetes medication (95%CI: 1.3–2.7) (Table 1). Considering the 3 criteria above, the prevalence of DM in the study population was 9.4% (Table 1).

**Table 1:** Sociodemographic and health conditions in adults according to the presence of diabetes. COVID-Inconfidentes (2020)

Characteristics	Total % (95%CI)	Without diabetes % (95%CI)	With diabetes % (95%CI)	*p*-value*
**Total**		90.6 (88.5–92.4)	9.4 (7.6–11.5)	-
Sociodemographic				
**Sex**				
Male	48.1 (41.0-55.2)	50.2 (42.4–58.0)	33.0 (22.3–45.7)	**0.027**
Female	51.9 (44.7–59.0)	49.8 (42.0-57.6)	67.0 (54.2–77.6)	
**Age**				
18 to 34 years	35.6 (31.1–40.3)	39.7 (33.4–42.7)	4.4 (1.2–5.7)	**< 0.001**
35 to 59 years	45.6 (41.1–50.2)	45.8 (42.4–51.9)	43.1 (31.6–59.2)	
≥ 60 years	18.8 (15.5–22.7)	14.5 (11.7–19.0)	52.5 (38.5–65.8)	
**Skin color** ^**a**^				
White	25.5 (20.8–31.2)	26.5 (20.9–32.9)	16.8 (10.5–23.6)	**0.006**
Black	20.2 (16.0-26.4)	19.4 (14.3–25.9)	34.7 (25.2–45.9)	
Brown	49.3 (41.5–54.4)	49.5 (42.3–56.5)	40.4 (31.5–50.9)	
Others	5.0 (4.1–7.8)	4.6 (3.4–5.9)	8.1 (3.9–16.7)	
**Marital status** ^**b**^				
Married Not married	54.0 (47.2–59.2)	52.7 (46.2–59.9)	58.2 (46.7–69.2)	0.405
	46.0 (40.8–52.8)	47.3 (40.1–53.8)	41.8 (30.8–53.1)	
**Education**				
0 to 8 years	31.2 (26.7–36.0)	27.7 (22.0-32.9)	58.6 (47.3–68.6)	**< 0.001**
9 to 11 years	39.7 (35.6–43.9)	40.9 (36.4–45.9)	27.4 (19.1–37.3)	
≥ 12 years	29.1 (23.8–35.1)	31.4 (25.8–38.4)	14.0 (8.8–22.5)	
**Family Income** ^**c**^				
≤ 2 MW	43.0 (35.6–46.8)	44.7 (39.5–49.3)	50.6 (40.2–61.9)	0.615
> 2 to ≤ 4 MW	31.9 (26.9–37.5)	30.4 (25.4–36.1)	27.3 (18.2–36.6)	
> 4 MW	25.1 (22.0-32.5)	24.9 (20.1–31.0)	22.1 (13.9–34.3)	
Health conditions				
**Smoking**				
No	82.3 (78.6–86.7)	82.1 (77.1–86.0)	92.0 (84.2–96.4)	**0.021**
Yes	17.7 (13.3–21.4)	17.9 (14.0-22.9)	8.0 (3.6–15.8)	
**Alcohol consumption**				
No	41.7 (36.0-47.9)	39.9 (33.6–46.6)	63.4 (52.7–73.2)	**< 0.001**
Yes	58.3 (52.1–64.0)	60.1 (53.3–66.4)	36.6 (26.8–47.3)	
**Nutritional status** ^**h**^				
BMI, kg/m²#	26.6 (26.2–27.0)	26.2 (25.9–26.6)	29.7 (27.9–31.4)	**< 0.001**
Underweight	2.5 (2.0-4.1)	2.6 (1.6–3.7)	4.6 (2.2–9.5)	
Eutrophic	41.9 (34.7–47.5)	44.1 (36.4–51.4)	22.3 (14.7–32.4)	**< 0.001**
Overweight	37.0 (29.5–44.9)	37.3 (29.3–46.4)	29.0 (18.2–43.1)	
Obesity	18.6 (15.9–23.0)	16.0 (13.2–20.0)	44.0 (29.4–59.8)	
**Physical activity** ^**i**^				
Physically active	30.3 (26.2–35.8)	32.9 (27.7–38.8)	14.1 (9.0-21.2)	**< 0.001**
Physically inactive	69.7 (64.2–73.7)	67.1 (61.2–72.3)	85.9 (78.8–91.0)	
**Sleep quality** ^***f***^				
Good	52.3 (48.6–56.4)	48.1 (44.0-52.8)	38.2 (27.4–49.0)	0.101
Poor	47.7 (43.6–51.4)	51.9 (47.2–56.0)	61.8 (51.0-42.6)	
**Vitamin D**				
≥ 20	80.1 (75.0-84.4)	81.3 (76.3–85.4)	68.6 (53.9–80.3)	**0.027**
< 20	19.9 (15.6–25.0)	18.7 (14.6–23.7)	31.4 (19.7–46.1)	
**FokI genotype**				
FF and Ff	54.7 (48.8–60.4)	55.7 (49.3–61.9)	44.6 (35.7–53.9)	0.062
Ff	45.3 (39.6–51.2)	44.3 (38.1–50.7)	55.4 (46.1–64.3)	
**Vitamin D supplementation**				
No	93.1 (91.8–95.3)	93.6 (91.6–95.3)	92.9 (85.1–96.4)	0.800
Yes	6.9 (4.7–8.2)	6.3 (4.7–8.4)	7.1 (3.6–14.9)	
**Exposure to sunlight** ^***k***^				
Sufficient	63.8 (59.0-70.4)	66.3 (60.4–72.3)	50.6 (37.8–62.4)	**0.019**
Insufficient	36.2 (29.6–41.0)	33.7 (27.6–39.6)	49.4 (37.6–62.2)	

MW: Minimum wage.

Vitamin D concentrations were classified according to the Institute of Medicine as deficient when 25(OH)D < 20 ng/mL; and sufficient when 25(OH)D ≥ 20 ng/mL.

# Presented mean and 95% confidence interval.

* *p*-value of Pearson’s chi-square test

a The participants were categorized into those with white, black, brown, and others race/skin colors (indigenous and yellows).

b Not married: Widowed, divorced, single

c Minimum wage value: BRL 1,045.00 ≈ USD 194.25 (1 USD = 5.3797 BRL)

f Poor sleep quality determined by PSQI ≥ 5.

h Underweight (BMI < 18.5 kg/m² if < 60 years or BMI < 22.0 kg/m² if > 60 years), eutrophic (BMI 18.5–24.9 kg/m² if < 60 years or BMI 22.0–27.9 kg/m² if > 60 years), overweight (BMI 25.0–29.9 kg/m² if < 60 years or BMI 28.0–29.9 kg/m² if > 60 years), obese (BMI > 30.0 kg/m²).

i Physically inactive (< 150 min/week of moderate physical activity or < 75 min/week of vigorous activity).

k Insufficient exposure to sunlight (< 30 min/day) and sufficient (≥ 30 min/day).

### Distribution of FokI polymorphism

Regarding the FokI polymorphism (rs2228570), the genotype frequency was 9.9% (95%CI: 5.8–16.3) for FF, 44.8% (95%CI: 41.0-49.1) for Ff, and 45.3% (95%CI: 39.3–51.0) for ff. The genotype distributions for the FokI polymorphism did not deviate from the expectations predicted by HWE (*p* > 0.05), as determined by a chi-square test in both groups (Table 1).

### Vitamin D, *VDR* gene polymorphism, and DM

The increased prevalence of diabetes is associated with the presence of the mutated allele. The results showed that individuals with the FF genotype had a prevalence of diabetes mellitus (DM) of 3.9%, while those with the Ff genotype had a prevalence of 8.5%. Notably, individuals with the ff genotype showed the highest prevalence of DM, reaching 11.5%. The coexistence of the ff genotype with vitamin D deficiency resulted in an even higher prevalence of DM, reaching 20.4% (Fig. 2b).

In the multivariate model, an association was observed between vitamin D deficiency (< 20 ng/mL) and DM. Individuals with vitamin D deficiency have a 2.19 times more chance (95% CI: 1.06–4.50) to have DM. Similarly, in the adjusted model, an association was observed between the FokI polymorphism and DM. It was observed that individuals with the altered allele (ff) had a 1.78 higher prevalence of DM (OR: 1.78; 95% CI; 1.10–2.87) (Table 2).

**Table 2:** Association between vitamin D levels, FokI polymorphism and diabetes

Variables		Unadjusted OR (95%CI)	*p*	Adjusted* OR (95%CI)	*p*
Vitamin D levels	≥ 20	1.00		1.00	
	< 20	1.96 (1.07–3.68)	**0.030**	2.19 (1.06–4.50)	**0.033**
FokI genotype	FF and Ff	1.00		1.00	
	Ff	1.56 (0.98–2.50)	0.062	1.78 (1.14–2.81)	**0.012**

OR: Odds ratio; CI: Confidence interval.

*The directed acyclic graph (DAG) was used to support the theoretical model for the adjusted analysis between FokI polymorphism (explanatory variable) and diabetes (outcome). Adjusted analysis by the following minimum and sufficient set of variables: sex, age, family income, body mass index, disease related to vitamin D metabolism and physical activity.

Collinearity among variables in the adjusted model evaluated by variance inflation factor (VIF) with the maximum remaining VIF = 1.1271

**Fig. 3:**
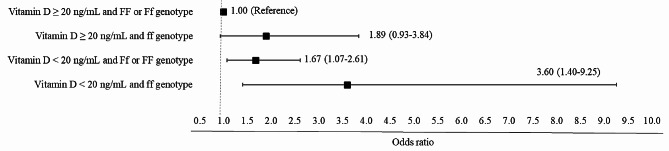
Association between vitamin D levels and the genotype of the FokI polymorphism with diabetes mellitus in adults. COVID-Inconfidentes study (2020) **Subtitle**: A multiplicative interaction analysis was performed to verify how vitamin D levels and the FokI polymorphism interfere with diabetes mellitus levels. The directed acyclic graph (DAG) was used to support the theoretical model for the adjusted analysis between the FokI polymorphism and vitamin D (explanatory variable) and diabetes mellitus (outcome). Analysis adjusted for the following minimum and sufficient set of variables: sex, age, family income, body mass index, diseases related to vitamin D metabolism and physical activity

Furthermore, combined analyses were conducted, by which it was possible to verify that individuals with vitamin D deficiency were associated with DM regardless of genotype (Ff + ff: OR: 1.67; 95%CI; 1.07–2.61; ff: OR: 3.60; 95% CI; 1.40–9.25). While individuals with the presence of the altered allele (ff) and vitamin D sufficiency were not associated with DM (Fig. 3).

## Discussion

This study aimed to investigate the association between diabetes with vitamin D deficiency and the VDR gene variant (FokI), which is related to the binding of vitamin D to its receptor [18]. To our knowledge, there are few studies that have explored the influence of vitamin D levels with VDR gene SNPs on the association with DM. Our findings suggested that vitamin D deficiency as well as the presence of the FokI polymorphism are associated with DM. Furthermore, combined analyses revealed that individuals with vitamin D deficiency were associated with DM regardless of genotype. While individuals with the presence of the altered allele (ff) and vitamin D sufficiency were not associated with DM. We emphasize the clinical relevance of these data for the management, control, and complications of DM.

Glucose metabolism is influenced by external (environment and lifestyle) and internal (genetic) factors. Studies have tried to understand what mechanisms could explain this pathophysiology. The development of type 2 DM involves impaired pancreatic β-cell function, insulin resistance, and inflammation [40]. The evidence surrounding vitamin D and glucose metabolism began with the discovery that β cells have vitamin D receptors and that in the absence of the vitamin D receptor and/or its deficiency, insulin secretion is impaired [14, 41, 42]. In addition, the immunomodulatory properties of 1,25(OH)2D3 are able to negatively regulate the production of inflammatory cytokines, contributing to reducing the risk of the development of type 2 DM [40].

The findings of the present study are consistent with the literature in which low serum vitamin D levels are associated with DM. A meta-analysis of observational and cross-sectional studies revealed that low levels of vitamin D were associated with an increased odds ratio of hyperglycemia in both diabetic and non-diabetic individuals. The authors suggested that vitamin D supplementation may be a strategy for the glycemic management of individuals [14]. In another prospective cohort study and meta-analysis, an association of low plasma 25(OH)D level with increased risk of type 2 DM was found. This finding was supported by the meta-analysis of prospective cohort and case-control studies [43]. In line with this, a literature review demonstrated a strong association between the FokI polymorphism and type 2 DM, pointing out that this gene polymorphism was possibly a risk factor for type 2 DM [41]. Furthermore, a systematic review and meta-analysis of randomized clinical trials investigating vitamin D supplementation combined with calcium showed positive effects on insulin, insulin resistance, and blood glucose. However, the authors emphasize caution with dosages, as these results were observed at high doses, and care needs to be taken to elucidate the appropriate amounts for different populations [9].

Moreover, there is evidence that the FokI polymorphism (the main mediator of vitamin D action) may affect insulin secretion and insulin resistance [44]. The mechanisms involved in the FokI polymorphism and the pathogenesis of DM may be related, since vitamin D exerts its effect only upon binding to the VDR, influencing its activity in target tissues [43]. The FokI polymorphism alters the structure of the VDR protein, resulting in the incorporation of three extra amino acids, which influences transcriptional activity by modulating the interaction with transcription factor IIB [45]. The ff allele, the polymorphic form, acts to increase the risks of pathological phenotypes [9, 17, 46–48]. In addition, some pathways such as calcium metabolism, inflammatory cytokine production, and adipocyte modulation may reinforce this susceptibility axis of FokI in the mechanism surrounding DM development, being pointed out as a gene that is involved in insulin secretion for insulin resistance [40].

There are few studies that have sought to investigate the association between vitamin D levels with the FokI polymorphism in the pathogenesis of DM. Our results revealed that the “ff” genotype of the FokI polymorphism is associated with DM, and in synergism with vitamin D deficiency, increases the likelihood of DM in adults. Furthermore, the ff genotype for the FokI polymorphism was found to be the most prevalent in the population and also the most frequent in diabetics. Our findings are supported by Mackawy and Badawi [40], Schuch, Garcia [44] and Ogunkolade, Boucher [45], the results of which showed that individuals homozygous recessive mutant (ff) had a significantly higher index of insulin resistance (HOMA-IR) than individuals with the heterozygous (Ff). In agreement, a meta-analysis suggested that the FokI polymorphism was associated with a significantly increased overall risk of type 2 DM [49]. Corroborating, in a study conducted on the Moroccan population, a significant association between FokI distribution and type 2 DM was reported [19]. In contrast, Malecki et al. [50] and Bid et al. (2009) [51] showed no significant associations between VDR (FokI) genotypes and diabetes risk. This inconsistency in findings may be explained by genetic differences in the populations or by external factors, such as environment and diet. Furthermore, our results highlight that although homozygous mutant (ff) individuals were more predisposed to have DM, VDD individuals were more prone regardless of phenotype, i.e. vitamin D deficiency is a relevant predictor for developing DM regardless of whether there is an alteration in the VDR receptor.

The results of this research provide relevant data for clinical and public health purposes, but have limitations that are worth mentioning: (i) The cross-sectional design does not allow us to assess causality. (ii) Residual confounding by non-measurable factors cannot be completely excluded. (iii) The study included participants from only two cities in Brazil. It is important to note that the population of the two cities is predominantly composed of black and brown people, due to the slavery past, and this may have an influence on vitamin D metabolism and present genetic variations. Therefore, large-scale genetic epidemiological studies including a greater diversity of ethnicities/races are needed for a better understanding of the association between vitamin D, FokI gene polymorphism, and DM. (iv) Although the FokI polymorphism has been reported as an independent marker of the VDR gene because it is not in linkage disequilibrium with any other VDR polymorphism [52, 53], it is important to recognize that linkage disequilibrium may be influenced by additional factors not considered in the current study. This includes other loci within the VDR gene that were not analyzed, as well as genetic factors external to the VDR gene that may affect linkage disequilibrium.

Our study has several strengths: there are few studies in the literature evaluating the genetic relationship between FokI polymorphism, vitamin D and DM in the world. Research in different populations, with specific and heterogeneous characteristics becomes crucial for genomic medicine, since most genetic studies are in European populations, while populations of African, Latin American, and Hispanic descent are poorly represented (less than 4% of all published research). Therefore, these data present low reproducibility and relevance to different ethnicities, and further associations with disease characteristics in other populations are needed [54]. The results of this study can serve as a basis for future research in other regions/populations, allowing for interpopulation comparisons and the development of personalized interventions for diabetes prevention and treatment in different contexts. Moreover, the present study is a novel evaluation of the Brazilian population. The study relies on a probability sample which provides statistical power to the study; a face-to-face population-based household study (conducted during the COVID-19 pandemic) and the use of the DAG to direct the analyses and avoid unnecessary adjustments.

The data shown represent a concern in terms of public health, both because of the high costs caused by DM to health systems, and the complications in the patient’s health. It is possible to note the increasing incidence of DM worldwide, in addition to the increasing prevalence of vitamin D deficiency. Against this background, our data support the creation of strategies for the prevention and management of the worsening of DM. The FokI polymorphism is a non-modifiable genetic factor; however, external factors such as adequate levels of vitamin D may contribute in a fundamental way to control blood glucose. Therefore, public policies that encourage increased sun exposure, consumption of natural and vitamin D-fortified foods, and the use of vitamin D supplements may be crucial to this alarming scenario. It is reinforced that the findings are recent, and the adequate doses of vitamin D are still uncertain, requiring future studies.

## Conclusion

Our findings suggested that individuals with vitamin D deficiency and the presence of the altered allele in homozygosisis (ff) of the FokI polymorphism (rs2228570) are more likely to have DM. Clinical applications should be careful, although it is considered a plausible intervention in the prevention, management, and treatment of DM, besides being a possible genetic marker for risk groups for diabetes mellitus.

### Electronic supplementary material

Below is the link to the electronic supplementary material.

Supplementary Material 1

## Data Availability

The datasets generated and/or analyzed as part of the current study are not publicly available due to confidentiality agreements with subjects. However, they can be made available solely for the purpose of review and not for the purpose of publication from the corresponding author upon reasonable request.
