# Supplementary material for: Vitamin D deficiency and VDR gene polymorphism FokI (rs2228570) are associated with diabetes mellitus in adults: COVID-inconfidentes study
Source: Diabetol Metab Syndr. 2024 May 30;16:118. doi: 10.1186/s13098-024-01328-6 (PMC11137993; doi:10.1186/s13098-024-01328-6)
Supplement: Supplementary file 1 — Supplementary Material 1 [file 13098_2024_1328_MOESM1_ESM.docx]

**Vitamin D deficiency and *VDR* gene polymorphism FokI (rs2228570) are associated with diabetes mellitus in adults: COVID-Inconfidentes Study**

Samara Silva de Moura^ab^, Luiz Antônio Alves de Menezes-Júnior^ab^, Ana Maria Sampaio Rocha^b^, Aline Priscila Batista^bc^, Thaís da Silva Sabião^ab^, Mariana Carvalho de Menezes^d^, George Luiz Lins Machado-Coelho^ab^, Júlia Cristina Cardoso Carraro^d^, Adriana Lúcia Meireles^d^

^a^ Universidade Federal de Ouro Preto, School of Nutrition, Postgraduate Program in Health and Nutrition, Research and Study Group on Nutrition and Public Health (GPENSC), Campus Morro do Cruzeiro, 35400-000 Ouro Preto, Minas Gerais, Brazil.

^b^ Epidemiology Laboratory, Medical School, Universidade Federal de Ouro Preto, Campus Morro do Cruzeiro, 35400-000 Ouro Preto, Minas Gerais, Brazil.

^c^ Universidade Federal de Ouro Preto, Postgraduate Program in Biological Sciences, Campus Morro do Cruzeiro, 35400-000 Ouro Preto, Minas Gerais, Brazil.

^d^ Universidade Federal de Ouro Preto, Department of Clinical and Social Nutrition, Research and Study Group on Nutrition and Public Health (GPENSC), School of Nutrition, Campus Morro do Cruzeiro, 35400-000 Ouro Preto, Minas Gerais, Brazil.

**Corresponding Authors:**

Adriana Lúcia Meireles

R. Diogo de Vasconcelos, 122, Ouro Preto- MG, Brazil.

Email: adriana.meireles@ufop.edu.br

**Funding:** This study was supported by the Federal University of Ouro Preto (UFOP) [PROPPI/UFOP nº03/2023], Brazilian Council for Scientific and Technological Development (CNPq), Coordination for the Improvement of Higher Education Personnel-Brazil (CAPES) [nº09/2020; CAPES-EPIDEMIAS 676/2020; nº88881.504995/2020-01], Foundation for Research Support of the State of Minas Gerais (FAPEMIG) [nº001/2021; APQ-02445-21], and finance code 001 for Ph.D. student scholarship.

**Declaration of competing interest:** The authors declare that they have no known competing financial interests or personal relationships that could have appeared to influence the work reported in this paper.

**Acknowledgments:** The authors acknowledge the support of the Federal University of Ouro Preto (UFOP) and the Research and Education Group in Nutrition and Collective Health (GPENSC) for their support and incentive, Clinical Analysis Pilot Laboratory of the Pharmacy School of the Federal University of Ouro Preto (UFOP) for the biochemical analyses, and also, the support of the Municipal Health Secretariats of the municipalities.

**Informed Consent Statement:** Informed consent was obtained from all subjects involved in the study.

**Institutional Review Board Statement:** All procedures adopted in this study followed the Declaration of Helsinki and the Brazilian guidelines and norms for research involving humans and were approved by the Research Ethics Committee of the Federal University of Ouro Preto, under protocol number 32815620.0.1001.5149.

**Data Availability Statement:** The datasets generated and/or analyzed as part of the current study are not publicly available due to confidentiality agreements with subjects. However, they can be made available solely for review and not for publication by the corresponding author upon reasonable request.
